# Frailty as an Independent predictor of perioperative risk and recovery in long-segment thoracolumbar fusion surgery: A retrospective cohort study

**DOI:** 10.1016/j.xnsj.2025.100749

**Published:** 2025-06-16

**Authors:** Ishav Y. Shukla, Sukul Mittal, Emerson Lout, Kristen Hall, Umaru Barrie, Omar S. Akbik, Salah G. Aoun, Carlos A. Bagley

**Affiliations:** a Department of Neurological Surgery, University of Texas Southwestern Medical Center, Dallas, TX, United States; b Department of Neurological Surgery, NYU Langone Health, New York, NY, United States; c Department of Neurological Surgery, Creighton University School of Medicine, Omaha, NE, United States; d Department of Neurological Surgery, Saint Luke’s Neurological & Spine Surgery, Kansas City, MO, United States

**Keywords:** Adult spinal deformity, Frailty, Long-segment thoracolumbar fusion, Modern frailty index-5, Perioperative outcomes, Risk stratification, Recovery, Spine surgery

## Abstract

**Background:**

Frailty, a multifactorial condition reflecting diminished physiological reserve, is associated with worse surgical outcomes and recovery, particularly in complex surgical procedures. This study aims to investigate frailty, as measured by the mFI-5, as a predictor of perioperative risk and recovery in patients undergoing long-segment thoracolumbar fusion for adult spinal deformity (ASD).

**Methods:**

We conducted a retrospective analysis to identify patients undergoing long-segment thoracolumbar fusion for ASD. Our primary outcome measures included anesthesia duration, estimated blood loss (EBL), intraoperative blood transfusion volumes, ICU stay duration, hospital length of stay (LOS), time to ambulation, postoperative complications, readmission rates, and discharge disposition. Patients were categorized as not frail (mFI-5 = 0), partially frail (mFI-5 = 1), or frail (mFI-5 ≥ 2). ANOVA and chi-square tests examined group differences, while multivariable regression, adjusting for age, gender, BMI, and number of levels fused, assessed frailty’s impact on perioperative outcomes.

**Results:**

About 235 patients were included; 45.1% were frail, 34.5% were partially frail, and 20.4% were not frail. In multivariable regression analysis, each unit increase in frailty score was associated with longer anesthesia duration (β = 11.1 minutes, p = .023), greater EBL (β = 150.5 mL, p = .026), and higher intraoperative blood transfusion volumes (β = 228.2 mL, p = .002). Frailty also independently predicted prolonged ICU stays (β = 9.0 hours, p = .002), increased hospital LOS (β = 0.6 days, p = .015), and delayed time to ambulation (β = 0.4 days, p = .039). Postoperative complications, readmission rates, and discharge disposition were not significantly different among frailty groups.

**Conclusions:**

Frailty independently predicts longer anesthesia duration, greater EBL, increased intraoperative transfusion requirements, and significantly delayed postoperative recovery in ASD patients, including prolonged ICU stays, extended hospital LOS, and delayed ambulation. While targeted perioperative strategies—such as optimizing preoperative comorbidities and enhancing intraoperative monitoring and rehabilitation—may not reduce a patient’s underlying frailty status, our findings suggest that early identification of frailty can help tailor perioperative planning, anticipate recovery trajectories, and support informed decision-making for clinicians and patients.

## Introduction

The increasing lifespan of the population presents significant challenges for orthopedic care, as aging individuals face a rising incidence of degenerative spinal disorders requiring surgical intervention [1]. Frailty is characterized by an age-related decline in the functioning of organ systems and a heightened susceptibility to adverse postoperative outcomes such as infection, the need for operation, and mortality [1]. Age closely correlates with frailty, contributing to unfavorable surgical outcomes [2]. To address this, accurate and effective prognostic indicators for perioperative outcomes in the frail population are crucial.

The 5-factor modified frailty index (mFI-5) is one such frailty index that assesses 5 patient factors: functional status, history of diabetes mellitus (insulin and noninsulin dependent), chronic obstructive pulmonary disease (COPD), congestive heart failure (CHF), and hypertension [3]. The mFI-5 has emerged as a shorter and simpler alternative to the 11-factor modified frailty index (mFI-11), demonstrating comparative effectiveness in the predictive value [4]. The mFI-5 has been validated across various surgical specialties and has been shown to be an independent predictor of adverse outcomes [5,6]. Notably, in orthopedic and neurosurgical surgeries, elevated mFI-5 scores are associated with complications, readmissions, and additional surgical procedures [[3], [4], [5],^7^,^8^].

Given the high prevalence of adult spinal deformity (ASD) in the aging population and the increased likelihood of comorbid conditions as individuals age, there is a pressing need for preoperative screening and risk stratification. While prior studies have highlighted the importance of frailty in predicting surgical outcomes for ASD [4,6], our study expands this understanding by incorporating less frequently examined variables, including preoperative hemodynamic stability, intraoperative transfusion requirements, readmissions, and discharge disposition, alongside traditionally studied metrics such as anesthesia duration, estimated blood loss, and complications.

Additionally, we provide a unique perspective by examining both frailty group classifications and the impact of each unit increase in mFI-5 scores. This granular approach enabled us to quantify how incremental increases in frailty correlate with perioperative outcomes, offering deeper insight into the role of frailty in ASD surgery. By examining frailty as a multifactorial construct and its association with a wide range of perioperative factors, we aim to assess its role in risk stratification and perioperative management for patients undergoing long-segment thoracolumbar fusion for ASD. We hypothesize that increasing frailty status will be associated with worse perioperative outcomes and delayed recovery.

## Methods

### Study design

We performed a retrospective cohort study assessing patients who underwent surgical correction of ASD at a single, academic, tertiary care medical institution between January 1, 2016, and January 7, 2021. The study protocol was approved by the Institutional Review Board, and a waiver of informed consent was granted as the study was considered to pose minimal risk to patients. This study adheres to the STROBE (Strengthening the Reporting of Observational Studies in Epidemiology) guidelines, ensuring comprehensive and transparent reporting of study design, methodology, and results [9].

### Patient selection

The study utilized ICD 9/10 and Current Procedural Terminology codes to identify all long segment (≥4 levels) lumbar and thoracolumbar fusions performed for spinal deformities. Preoperative imaging, including X-rays and CT scans, was reviewed by neurosurgeons (O.A., S.G.A., and C.A.B.) to confirm the presence and laterality of deformity, as well as the spinal parameter measurements (Cobb angle, sagittal vertical axis, sacral slope, pelvic tilt, and pelvic incidence). Surgical candidacy was determined using a combination of radiographic thresholds (eg, Cobb angle >20°, SVA >5 cm) and patient-reported outcomes, including Visual Analog Scale (VAS) scores, functional assessments by occupational therapy (OT), and PROMIS-29 questionnaire data.

Patients without at least a 4-level lumbar segment fusion, preoperative mFI-5 scores, or adequate perioperative data were excluded from the study. All surgeries were performed by the senior author (C.A.B.), with surgical indication and fusion extent determined at the surgeon’s discretion based on the combination of radiographic deformity and symptomatic burden. The majority of procedures were performed via a posterior-only approach; however, a small subset of patients (*n* = 6) underwent anterior lumbar interbody fusion as part of a staged anterior-posterior approach.

### Data collection

In total, 235 patients met the inclusion criteria. The electronic medical record of each patient was independently reviewed to extract target pre-, intra-, and postoperative patient data. Preoperative data included demographics (age and gender), body mass index (BMI), comorbid conditions (eg, hypertension, chronic obstructive pulmonary disease [COPD], obstructive sleep apnea), American Society of Anesthesiologists (ASA) physical status classification, and mFI-5 scores, which were systematically derived through retrospective chart review using standardized criteria.

The mFI-5 was selected as the frailty assessment tool for this study due to its robust predictive value in perioperative outcomes and its adaptability to data from the American College of Surgeons—National Surgical Quality Improvement Program (ACS-NSQIP). Originally derived from the mFI-11, the mFI-5 was developed to include variables that are consistently and accurately recorded, addressing limitations of the mFI-11, which has seen reduced reliability due to missing or inconsistently recorded data [7,10]. The mFI-5 has been validated across a wide range of diagnoses and surgical procedures and has demonstrated effectiveness in predicting perioperative outcomes. Frailty was classified into 3 categories based on mFI-5 scores: not frail (NF, mFI-5 = 0), partially frail (PF, mFI-5 = 1), and frail (F, mFI-5 ≥ 2), following established literature [7,11].

Furthermore, electronic medical records were examined to document date of surgery, intraoperative variables (eg, duration of anesthesia, estimated blood loss, and transfusion volumes), and postoperative outcomes (eg, intensive care unit (ICU) length of stay (LOS), total hospital LOS, time to ambulation, discharge location, and readmissions at 7-, 30-, and 90-days). Intraoperative outcomes and postoperative recovery metrics were calculated from the thoracolumbar fusion (posterior stage) only, regardless of whether patients underwent a staged anterior-posterior approach.

For all patients, the following postoperative complications were evaluated: hardware failure (eg, broken rod, disconnected iliac screw), proximal junctional kyphosis (PJK), pseudoarthrosis, wound infection and others (seroma, chronic pain, adjacent segment disease, and hematoma). Although "other" complications were included, the sample size for these events was too small to allow for meaningful statistical analyses. PJK was defined radiographically as a sagittal Cobb angle ≥10° between the upper instrumented vertebra and the vertebra 2 levels above, with an increase of at least 10° from the preoperative measurement, consistent with established criteria [12,13]. Clinical findings such as new or worsening pain at the proximal junction or the development of new neurological deficits were also utilized as adjunctive indicators.

### Statistical analysis

Data were analyzed using SPSS Statistics Version 29.0 (IBM Corp., Armonk, NY). Continuous variables were summarized as means with standard deviations (SDs) to enhance readability and to provide a comprehensive representation of population variation. Welch’s t-test was used to compare means of continuous variables between 2 groups, while analysis of variance (ANOVA) evaluated differences across frailty. For significant ANOVA results, post hoc pairwise comparisons were conducted to identify specific group differences. Categorical variables were summarized as frequencies and percentages, and associations between categorical variables were analyzed using Chi-square tests or Fisher’s exact tests. Multivariable linear regression was performed using the raw mFI-5 score as a continuous predictor to enable greater granularity in assessing its relationship with continuous outcomes (eg, anesthesia duration, estimated blood loss, length of stay).

Logistic regression models, however, utilized the frailty categories (not frail, partially frail, frail) to assess their association with binary outcomes (eg, presence of postoperative complications, 30-day readmission, discharge location). This dual approach allowed for both detailed and clinically interpretable findings. All multivariable regression models were adjusted a priori for age, gender, BMI, and the number of levels fused. For all regression analyses, beta coefficients (for linear regression) or odds ratios (ORs) (for logistic regression), along with 95% confidence intervals (CIs), were reported. Statistical significance was defined as a p-value ≤ .05.

## Results

### Baseline demographics, comorbidities, and frailty-associated differences

Baseline demographic, clinical, and comorbidity characteristics are summarized in Table 1. The study cohort included 235 patients, with a mean age of 69.6 years. Female patients comprised 66.0% of the cohort. The mean BMI was 28.2 kg/m², and 49 patients (20.9%) met criteria for obesity class 1 (BMI 30–34.9), 22 (9.4%) for obesity class 2 (BMI 35–39.9), and 10 (4.3%) for obesity class 3 (BMI ≥ 40). The most common comorbidities were hypertension (58.7%), diabetes mellitus type II (16.2%), and obstructive sleep apnea (17.9%). Regarding ASA classification, 90 patients (38.3%) were classified as ASA class 2, 139 (59.1%) as ASA class 3 and 6 (2.6%) as ASA class 4.

**Table 1:** Baseline demographic, clinical, and comorbidity characteristics stratified by frailty categories.

Variable	Not Frail (mFI-5 = 0)	Partially frail (mFI-5 = 1)	Frail (mFI-5 ≥ 2)	Full cohort	p-value
Total number of patients	48 (20.4)	81 (34.5)	106 (45.1)	235	-
Demographics					
Age (years)	69.4 (7.3)	69.6 (8.7)	69.7 (7.8)	69.6 (8.0)	.749
Gender: Female	32 (66.7)	54 (66.7)	69 (65.1)	155 (66.0)	.968
BMI (kg/m^2^)	25.8 (4.7)	27.5 (5.7)	29.8 (6.6)	28.2 (6.2)	< .001*
Obesity					
Class 1 (BMI 30–34.9)	6 (12.5)	14 (17.3)	29 (27.4)	49 (20.9)	.145
Class 2 (BMI 35–39.9)	2 (4.2)	3 (3.7)	17 (16.0)	22 (9.4)	.008*
Class 3 (BMI ≥ 40)	0 (0)	3 (3.7)	7 (6.6)	10 (4.3)	.115
Smoking status					
Former smoker	11 (30.6)	21 (27.6)	50 (47.6)	82 (37.8)	.015*
Current smoker	1 (2.8)	0 (0)	4 (3.8)	5 (2.3)	.198
Comorbidities					
Hypertension	1 (2.1)	46 (56.8)	91 (85.8)	138 (58.7)	< .001*
Osteopenia	9 (18.8)	13 (16.0)	21 (19.8)	43 (18.3)	.461
Diabetes mellitus type 2	0 (0)	3 (3.7)	35 (33.0)	38 (16.2)	< .001*
Obstructive sleep apnea	9 (18.8)	12 (14.8)	21 (19.2)	42 (17.9)	.621
Osteoporosis	8 (16.7)	11 (13.6)	18 (17.0)	35 (16.1)	.784
COPD	1 (2.8)	2 (2.5)	22 (20.8)	25 (10.6)	< .001*
Coronary artery disease	0 (0)	6 (7.4)	14 (13.2)	20 (8.5)	.036*
Chronic kidney disease	4 (8.3)	6 (7.4)	7 (6.6)	17 (7.2)	.938
Congestive heart failure	0 (0)	1 (1.2)	7 (6.7)	8 (3.4)	.115
ASA					
Class 2	28 (58.3)	34 (42.0)	28 (26.4)	90 (38.3)	.001*
Class 3	20 (41.7)	45 (55.6)	74 (69.8)	139 (59.1)	.005*
Class 4	0 (0)	2 (2.5)	4 (3.8)	6 (2.6)	.756

Abbreviations: ASA, American Society of Anesthesiologists; BMI, Body Mass Index; COPD, chronic obstructive pulmonary disease; GERD, gastroesophageal reflux disease; PJK, proximal junctional kyphosis.

All categorical variables are represented in frequency (proportion).

All continuous variables are represented in mean (standard deviation).

⁎ Indicates statistical significance (p < .05).

Several variables demonstrated statistically significant differences among frailty categories. Frail patients had a mean BMI of 29.8 kg/m² compared to 27.5 kg/m² in the partially frail group and 25.8 kg/m² in the not frail group (p < .001). Similarly, the prevalence of obesity class 2 was higher in the frail group (16.0%) relative to the partially frail (3.7%) and not frail (4.2%) groups (p = .008). Hypertension was significantly more prevalent in frail patients (85.8%) compared to partially frail (56.8%) and not frail (2.1%) groups (p < .001). Diabetes mellitus type II was also more common in frail patients (33.0%) than in the partially frail (3.7%) and not frail (0%) groups (p < .001). COPD was present in 20.8% of frail patients compared to 2.5% of partially frail and 2.8% of not frail patients (p < .001). Coronary artery disease was reported in 13.2% of frail patients, 7.4% of partially frail patients, and was absent in the not frail group (p = .036). Smoking history differed significantly across frailty categories (p = .015), with 47.6% of frail patients, 27.6% of partially frail patients, and 30.6% of not frail patients classified as former smokers.

Frailty classification also correlated with ASA status. ASA class 3 (severe systemic disease that is not life-threatening) was observed in 69.8% of frail patients, 55.6% of partially frail patients, and 41.7% of not frail patients (p = .005). In contrast, ASA class 2 (mild systemic disease) was more common in the not frail group (58.3%) compared to 42.0% in the partially frail group and 26.4% in the frail group (p = .001). No other demographic or clinical variables demonstrated statistically significant differences among frailty categories.

### Perioperative characteristics and postoperative outcomes

Perioperative characteristics and outcomes stratified by frailty categories are summarized in Table 2. The mean Cobb angle for the full cohort was 25.6°, with a sagittal vertical axis of 5.8 mm, sacral slope of 27.8°, pelvic tilt of 23.5°, and pelvic incidence of 49.5°. The mean number of levels fused was 9.3. None of these spinal parameters were significantly different across frailty categories.

**Table 2:** Perioperative parameters and outcomes stratified by frailty categories.

Variable	Not frail (mFI-5 = 0)	Partially frail (mFI-5 = 1)	Frail (mFI-5 ≥ 2)	Full cohort	p-value
Spinal parameters					
Cobb angle	26.6 (11.4)	27.8 (12.8)	23.2 (13.9)	25.6 (15.5)	.067
Sagittal vertical axis	6.2 (5.1)	5.3 (4.0)	7.1 (4.9)	5.8 (5.9)	.102
Sacral slope	28.7 (10.4)	28.7 (10.8)	29.8 (10.6)	27.8 (13.7)	.483
Pelvic tilt	22.4 (9.1)	23.8 (8.4)	24.6 (10.5)	23.5 (12.5)	.599
Pelvic incidence	47.7 (13.8)	49.8 (12.3)	51.6 (12.3)	49.5 (16.1)	.192
Numbers of levels fused	9.2 (2.9)	9.0 (2.6)	9.6 (2.9)	9.3 (2.8)	.255
Preoperative lab measurements					
Hemoglobin	13.4 (1.1)	13.3 (1.5)	13.0 (1.7)	13.2 (1.5)	.169
Hematocrit	40.3 (2.8)	40.0 (4.2)	39.1 (4.8)	39.7 (4.3)	.114
Platelets	231.3 (59.3)	244.7 (58.7)	241.1 (77.0)	240.3 (67.6)	.308
BUN	17.4 (6.4)	19.8 (9.7)	17.8 (7.4)	18.4 (8.1)	.103
Creatinine	1.4 (3.6)	2.0 (10.3)	1.0 (0.7)	1.4 (6.3)	.439
Surgical operative parameters					
Anesthesia duration (min)	280.2 (47.0)	288.5 (69.1)	301.4 (81.9)	292.6 (71.8)	.247
Estimated blood loss (mL)	1342.1 (671.4)	1603.9 (980.0)	1693.6 (1079.5)	1590.4 (978.9)	.237
Intraoperative transfusions (mL)					
Blood	735.0 (722.4)	1013.0 (949.0)	1194.4 (1221.8)	1038.1 (1054.9)	.057
Fresh frozen plasma	181.2 (321.5)	286.8 (425.9)	357.7 (575.1)	297.2 (485.4)	.158
Platelets	37.2 (89.1)	33.0 (92.7)	58.1 (125.7)	45.2 (108.4)	.361
Postoperative complications					
Any complication	17 (35.4)	32 (39.5)	34 (32.1)	83 (35.3)	.416
Hardware failure	2 (5.6)	8 (9.9)	14 (13.2)	26 (11.1)	.460
Proximal junctional kyphosis	5 (10.4)	8 (9.9)	7 (6.6)	20 (8.5)	.581
Pseudoarthrosis	7 (14.6)	5 (6.2)	7 (6.6)	19 (8.1)	.483
Wound infection	2 (4.2)	4 (4.9)	4 (3.8)	10 (4.3)	.902
Hospital stay and recovery					
ICU length of stay (hours)	38.0 (28.2)	36.3 (19.6)	46.9 (54.0)	41.4 (40.1)	.513
Total length of stay (days)	5.9 (1.9)	6.0 (2.6)	6.6 (4.1)	6.3 (3.3)	.660
Time to ambulation (days postsurgery)	1.9 (1.1)	2.1 (1.8)	2.5 (3.6)	2.3 (2.7)	.934
Readmissions					
7-day	0 (0)	2 (2.5)	3 (2.8)	5 (2.1)	.512
30-day	2 (4.2)	7 (8.6)	10 (9.4)	19 (8.1)	.526
90-day	3 (6.3)	12 (14.8)	19 (17.9)	34 (14.5)	.161
Discharge disposition					
Home	16 (33.3)	30 (37.0)	37 (34.9)	83 (35.3)	.881
Assisted living	5 (10.4)	3 (3.7)	10 (9.4)	18 (7.7)	.259
Rehabilitation	25 (52.1)	39 (48.1)	49 (46.2)	113 (48.1)	.793

Abbreviations: BUN**,** blood urea nitrogen; ICU, intensive care unit.

All categorical variables are represented in frequency (proportion).

All continuous variables are represented in mean (standard deviation).

Preoperative laboratory values, including hemoglobin, hematocrit, platelet count, BUN, and creatinine, were not statistically significantly different among the frailty groups. Intraoperative transfusion volumes were consistently higher in frail patients compared to the other groups, although these differences were not statistically significant. Mean blood transfusion volume was highest in frail patients (1,194 mL) compared to 1,013 mL in partially frail and 735 mL in not frail patients. Similarly, fresh frozen plasma transfusion volume was highest in frail patients (358 mL) compared to 287 mL in partially frail and 181 mL in not frail patients. Platelet transfusion volumes followed the same trend, with frail patients receiving an average of 58 mL, compared to 33 mL in partially frail and 37 mL in not frail patients.

Postoperative complication rates were comparable across frailty groups, with any complication occurring in 35.3% of the overall cohort. The incidence of hardware failure was highest in frail patients (13.2%) compared to 8.9% in partially frail and 5.6% in not frail patients. PJK was observed in 6.6% of frail patients, 9.9% of partially frail patients, and 10.4% of not frail patients. Pseudoarthrosis rates were 7.6% in frail patients, 6.2% in partially frail, and 14.6% in not frail patients, while wound infection rates remained low across all groups (frail: 3.8%, partially frail: 4.9%, not frail: 4.2%). None of these differences were statistically significant.

### Recovery metrics, readmission, and discharge outcomes

Hospital stays and recovery metrics demonstrated notable trends. ICU length of stay was longest in frail patients (46.9 hours) compared to 36.3 hours in partially frail and 38.0 hours in not frail patients. Total hospital length of stay increased with frailty, with frail patients staying an average of 6.6 days, partially frail patients 6.0 days, and not frail patients 5.9 days. Time to ambulation was progressively delayed with increasing frailty, with frail patients ambulating at 2.5 days postoperatively, compared to 2.1 days in partially frail and 1.9 days in not frail patients. However, none of these differences reached statistical significance.

Readmission rates trended higher in frail patients at both 30 days (9.4%) and 90 days (17.9%), compared to 7.6% and 14.8% in partially frail patients, and 4.2% and 6.3% in not frail patients, respectively. Seven-day readmission rates remained low across all groups. Discharge disposition patterns varied based on frailty status. Frail patients were discharged home (37.4%) compared to 37.0% in partially frail and 33.3% in not frail patients. Conversely, a higher proportion of frail patients required discharge to rehabilitation (46.2%), compared to 48.1% of partially frail and 52.1% of not frail patients. Discharge to assisted living was similar across groups, with 16.0% of frail patients, 14.8% of partially frail patients, and 14.6% of not frail patients requiring assisted living placement. None of these differences were statistically significant. No differences in discharge disposition across frailty groups reached statistical significance.

### Multivariable regression analysis of frailty and perioperative characteristics

Multivariable regression analysis was performed with Not Frail (mFI-5 = 0) as the reference group and controlling for age, gender, BMI, and the number of levels fused (Table 3). Among preoperative variables, each unit increase in mFI-5 score was associated with a 0.2 g/dL decrease in hemoglobin (β = −0.2, p = .034) and a 0.6% decrease in hematocrit (β = −0.6, p = .044). No significant associations were observed between frailty and platelet count, BUN, or creatinine levels. For surgical parameters, each unit increase in mFI-5 was associated with an increase of 11.1 minutes in anesthesia duration (β = 11.1, p = .023) and an increase of 150.5 mL in estimated blood loss (β = 150.5, p = .026).

**Table 3:** Multivariable regression analysis of mFI-5 and perioperative characteristics.

Variables	Beta or OR	95% CI	p-value
Preoperative variables			
Hemoglobin	−0.2	−0.4; −0.02	.034*
Hematocrit	−0.6	−1.2; −0.02	.044*
Platelets	2.1	−7.0; 11.3	.647
BUN	0.1	−1.0; 1.3	.821
Creatinine	−0.2	−1.1; 0.7	.677
Intraoperative variables			
Anesthesia duration (minutes)	11.1	1.5; 20.7	.023*
Estimated blood loss (mL)	150.5	17.9; 283.0	.026*
Transfusion volumes (mL)			
Blood transfusion	228.2	84.7; 371.6	.002*
FFP transfusion	105.0	38.6; 171.3	.002*
Platelet transfusion	13.9	0; 28.9	.070
Postoperative complications			
Any complication	0.7	0.3; 1.5	.689
Wound infection	0.8	0.1; 5.0	.835
Hardware failure	1.2	0.4; 4.1	.769
Pseudoarthrosis	0.4	0.1; 1.2	.107
Proximal junctional kyphosis	0.5	0.2; 1.9	.349
Length of stay and recovery			
ICU length of stay (hours)	9.0	3.4; 14.6	.002*
Total length of stay (days)	0.6	0.1; 1.0	.015*
Initiation of walking (days)	0.4	0.02; 0.8	.039*
Readmission			
30-d	2.5	0.5; 12.4	.257
90-d	3.4	0.9; 12.4	.064
Discharge location			
Assisted living	0.7	0.2; 2.4	.581
Home	1.2	0.6; 2.6	.627
Rehabilitation	0.7	0.3; 1.4	.299

Abbreviations: FFP, fresh frozen plasma; ICU, intensive care unit.

All continuous outcome variables are represented with the Beta Coefficient.

All binary outcome variables are represented with the Odds Ratio.

For all analysis, Not frail (mFI-5 = 0) was the reference group.

⁎ Indicates statistical significance (p < .05)

Regarding intraoperative transfusions, each unit increase in mFI-5 score was associated with an increase of 228.2 mL in blood transfusion volume (β = 228.2, p = .002) and an increase of 105.0 mL in fresh frozen plasma (FFP) transfusion volume (β = 105.0, p = .002). Platelet transfusion volumes also trended higher with frailty, but this association did not reach statistical significance (p = .070).

For postoperative outcomes, each additional frailty point was associated with a 9.0-hour increase in ICU length of stay (β = 9.0, p = .002) and a 0.6-day increase in total hospital length of stay (β = 0.6, p = .015). Additionally, frailty was significantly associated with a 0.4-day delay in time to ambulation (β = 0.4, p = .039). Although readmission rates trended higher with frailty, the associations did not reach statistical significance. Frail patients had 2.5 times the odds of 30-day readmission (OR = 2.5, p = .257) and 3.4 times the odds of 90-day readmission (OR = 3.4, p = .064) compared to not frail patients. Frailty was not significantly associated with discharge disposition to home, assisted living, or rehabilitation.

## Discussion

### The aging population and frailty

The global population over 65 years of age is projected to rise from 9% in 2019 to 16% by 2050, presenting significant challenges to healthcare systems, particularly in addressing the needs of frail patients [14]. Aging is associated with a higher burden of comorbidities, including osteoporosis, impaired mobility, spinal degeneration, and deformities, which contribute to the increasing prevalence of spinal disease [[14], [15], [16]].

Frailty, a multifactorial condition marked by diminished physiological reserve, emerges with age-related declines in multiple systems, increasing susceptibility to complications, disability, and mortality [[17], [18], [19]]. The prevalence of frailty rises with advancing age, affecting up to 26% of adults over 85 years [20], although it can manifest at any age [21,22]. Approximately one-third of all operating room-based procedures are performed on adults greater than 65 years of age and a significant portion of these individuals are considered frail [23]. Given its association with adverse outcomes, frailty has become an essential focus in surgical patients, particularly in procedures with high physiologic demands, such as long-segment thoracolumbar fusion for ASD correction.

### Key findings and significance

Our study highlights the significant impact of frailty on the perioperative and postoperative course of patients undergoing surgical correction for ASD. As expected, given the components of the mFI-5, frail patients had higher rates of hypertension, diabetes mellitus type II, and COPD. They also more frequently had comorbidities not captured by the mFI-5, such as obesity and coronary artery disease, and were more likely to be classified as ASA class 3 or higher, indicating severe systemic disease.

In our cohort, each unit increase in frailty scores, even after controlling for variations in age, gender, BMI, and the number of levels fused, independently predicted several key perioperative outcomes, including longer anesthesia duration, higher estimated blood loss, and increased intraoperative transfusion requirements, underscoring its association with greater surgical complexity. These findings may reflect challenges due to poor tissue quality and slower instrumentation, though these mechanisms remain speculative and warrant further investigation in future research.

Frailty was significantly associated with greater intraoperative transfusion requirements, with each unit increase in mFI-5 score correlating with an additional 228.2 mL of blood transfusion and 105.0 mL of fresh frozen plasma transfusion. Given that perioperative transfusions have been linked to increased risk of postoperative infections, cardiopulmonary complications, and prolonged ICU stays, frailty-associated transfusion requirements may contribute to downstream morbidity [[24], [25], [26]]. While causality cannot be inferred, these findings support the need for further research into preoperative optimization and transfusion practices in surgical candidates classifying as frail.

Interestingly, our observed PJK rate was on the lower end compared to previously published literature, which often reports rates between 20% and 40% [27]. This may be attributable to specific surgical techniques employed at our institution to minimize junctional stress. These include inserting the proximal pedicle screws parallel to the endplate to reduce rostral screw migration, undersizing the proximal screw diameter to allow for stress release at the screw-bone interface, selecting a lower entry point to avoid violation of the proximal facet joint, and ensuring that the screw tulip does not impinge upon the facet. Together, these technical strategies may have contributed to the reduced incidence of PJK in our cohort.

While frailty was not significantly associated with postoperative mechanical complications in our cohort, it independently predicted several delayed recovery metrics. Each unit increase in frailty corresponded to extended ICU length of stay (9 hours), total hospital length of stay (0.6 days), and delayed time to ambulation (0.4 days). While these absolute differences may appear limited on an individual level, they may still be clinically meaningful when considered cumulatively or at the population level—particularly in high-volume centers or resource-limited settings.

Additionally, these findings reinforce the critical need for early postoperative ambulation to reduce the risk of deep vein thrombosis, pulmonary complications, and prolonged functional decline in frail patients [[28], [29], [30]]. Additionally, a clinically meaningful trend toward higher readmission rates was observed, with frail patients having 2.5 times the odds of 30-day readmission and 3.4 times the odds of 90-day readmission, though these differences did not reach statistical significance.

The absence of statistically significant differences in complications and readmissions may be explained by several factors. First, our overall complication and readmission rates were relatively low, potentially limiting our power to detect differences across frailty groups. Second, the use of standardized perioperative protocols and consistent surgical technique at a single academic center may have mitigated frailty-associated risks. Finally, frailty may influence outcomes more subtly—manifesting as delayed functional recovery and increased resource utilization rather than overt complications—highlighting the importance of examining recovery metrics alongside traditional endpoints. While these results did not reach statistical significance, they suggest an increased risk of prolonged recovery and recurrent hospital visits in frail patients, emphasizing the need for enhanced discharge planning and outpatient follow-up care to prevent readmissions. Given the observed trends toward increased rehabilitation utilization and higher readmission rates, structured early mobility programs and enhanced rehabilitation coordination should be prioritized in frail patients to facilitate smoother recovery and reduce postdischarge complications.

### Clinical implications

Taken together, our findings demonstrate that frailty, as a multifactorial marker of systemic vulnerability, is a valuable tool for predicting outcomes and guiding perioperative management in high-risk patients. Crucially, each unit increase in frailty score significantly impacts the clinical course of patients undergoing long-segment thoracolumbar fusion, highlighting the need for targeted strategies to address the unique challenges faced by frail patients. Based on our findings, perioperative care should focus on optimizing preoperative comorbidities, reducing intraoperative burden, and implementing tailored postoperative recovery plans, including enhanced mobility programs and close postdischarge monitoring, to potentially mitigate the adverse effects of frailty. While frailty may not be fully modifiable, identifying it preoperatively provides clinicians, patients, and families with valuable insight into expected recovery trajectories and helps guide risk-informed perioperative planning.

### Comparison with prior studies

Our findings align with prior research demonstrating that frailty is a critical predictor of surgical complexity and recovery outcomes across various surgical specialties. Frailty has been shown to significantly increase the risk of complications, prolonged hospital stays, and readmissions in orthopedic procedures [31], and it is associated with elevated mortality and morbidity in aortic aneurysm repair and gastrointestinal surgery [32,33] Shinall Jr. et al. [34] further identified frailty as a predictor of increased mortality across all levels of operative stress, including minor surgical procedures. These studies, in accordance with our findings, underscore the pervasive influence of frailty on surgical outcomes, emphasizing the need for targeted interventions to address this high-risk population and continued research on its impact.

In the context of ASD correction, prior studies have consistently highlighted the association between frailty and adverse outcomes. Miller et al. [35] demonstrated that frail patients undergoing ASD correction experienced increased rates of major complications, proximal junctional kyphosis, pseudoarthrosis, wound infections, reoperations, and longer hospital stays. Similarly, Li et al. [36] reported that frail patients had significantly higher intraoperative blood loss, longer hospital stays, and more frequent major postoperative complications compared to nonfrail patients. Unlike previous studies, we did not find significant differences in postoperative complications or readmission rates among frailty groups, which may reflect several factors.

Our study was conducted in a single academic institution with standardized perioperative protocols and postoperative care, which may have reduced variability and mitigated the impact of frailty on complications and readmissions. While previous studies have largely focused on complications and mortality, our study uniquely emphasizes recovery metrics, including time to ambulation and resource utilization such as ICU and total hospital stays. This broader focus provides a comprehensive understanding of frailty’s impact on the entire surgical and recovery continuum for ASD patients. By highlighting both intraoperative complexity and postoperative recovery challenges, our findings offer actionable insights to improve care pathways for high-risk, frail patients undergoing complex surgical procedures.

### The future of frailty assessment in surgical care

Future studies are necessary to determine which patient factors carry the most predictive value to construct a holistic frailty index. Although efforts are ongoing to develop a standardized and universally applicable frailty index, it is equally important to recognize that the criteria for frailty may vary by surgical specialty. For instance, osteoporosis may be a critical predictor of perioperative outcomes in spine surgery but may hold less relevance in gastrointestinal procedures. Given the multifaceted and dynamic nature of frailty, machine learning–based approaches may offer a powerful means to identify the most procedure-relevant predictors of adverse outcomes.

While some frailty-related factors may not be modifiable, the ability to preoperatively assess a patient’s frailty remains clinically valuable—both for developing personalized management plans and for informing patients and families about potential risks, expected recovery trajectories, and postoperative care needs. Ultimately, such information can help guide shared decision-making and ensure that perioperative strategies are appropriately aligned with the patient’s risk profile.

### Study limitations

This study has limitations that warrant consideration. First, its retrospective and single-institution design limit the generalizability of our findings to broader populations. While having a single surgeon for all cases reduces variability in surgical techniques, it also introduces potential bias and confines the applicability of our results to similar practice settings. Additionally, the relatively small sample size may have constrained the ability to detect certain significant associations. Furthermore, the inherent heterogeneity of ASD patients, including variations in medical comorbidities, bone health, activity levels, and recovery trajectories, adds complexity to interpreting the results and their broader applicability. Future studies should incorporate more comprehensive frailty indices and larger, multicenter cohorts to validate and expand upon our findings.

## Conclusion

Our study found that frailty, as measured by the mFI-5, independently predicts longer anesthesia duration, increased estimated blood loss, higher intraoperative transfusion requirements, prolonged ICU and hospital stays, and delayed time to ambulation in patients undergoing surgical correction for ASD. These findings emphasize the utility of the mFI-5 as a practical tool for assessing frailty and guiding perioperative management in this high-risk population. While targeted perioperative strategies—such as optimizing comorbidities, enhancing intraoperative monitoring, and implementing structured rehabilitation—may not alter a patient’s underlying frailty status, early identification can guide recovery planning, inform perioperative decision-making, and provide valuable insight into expected outcomes for clinicians, patients, and families. Recognizing frailty preoperatively enables risk-informed care and may help tailor interventions to improve outcomes in this vulnerable group.

## Funding

This research did not receive any specific grant from funding agencies in the public, commercial, or not-for-profit sectors.

## Declaration of competing interest

One or more authors declare potential competing financial interests or personal relationships as specified on required ICMJE-NASSJ Disclosure Forms.
